# Ipsilateral Foot Drop and Erectile Dysfunction After the Use of a Traction Table in Intramedullary Femur Fixation: A Case Report and Review of the Literature

**DOI:** 10.7759/cureus.61316

**Published:** 2024-05-29

**Authors:** Yazan T Almaghrabi, Mohammad H Nooh, Sara G Qadi

**Affiliations:** 1 Orthopedic Surgery, King Abdulaziz Medical City, Ministry of National Guard Health Affairs, Jeddah, SAU; 2 College of Medicine, King Saud bin Abdulaziz University for Health Sciences, Jeddah, SAU; 3 Orthopedic Surgery, King Abdulaziz Medical City/King Saud bin Abdulaziz University for Health Sciences, Jeddah, SAU

**Keywords:** intramedullary nail, foot drop, traction table, erectile dysfunction, neck of femur fractures

## Abstract

This report presents a case of ipsilateral foot drop and erectile dysfunction following the use of a traction table during intramedullary femur fixation. The patient, a 39-year-old male, underwent surgery for a femur fracture using an intramedullary nail and was positioned on a traction table during the procedure. Post-operatively, he developed foot drop and erectile dysfunction. Neurological examination revealed peroneal nerve injury as the likely cause of the foot drop. The erectile dysfunction was attributed to pudendal nerve injury. Various treatment options were considered, including physical therapy for foot drop and phosphodiesterase inhibitors for erectile dysfunction. In conclusion, this case underscores the importance of recognizing and addressing potential complications associated with traction table use in orthopedic procedures, particularly concerning neurological sequelae and sexual dysfunction.

## Introduction

Traction tables, often utilized alongside perineal posts, play a crucial role in orthopedic surgery for stabilizing and fixing lower limb fractures. These tables are essential for providing traction, counter-traction, and immobilization during surgical procedures [1]. Specifically, the perineal post serves as a vital component for counter-traction. However, improper use or prolonged compression from the table and perineal post can lead to various complications, particularly injuries to perineal soft tissues and nerves. Among the reported complications are injuries to important nerves like the sciatic and pudendal nerves [2]. Such injuries can result from inappropriate positioning techniques, excessive pressure, or prolonged traction, highlighting the need for meticulous attention to patient positioning and table setup during orthopedic procedures.

## Case presentation

This is a 37-year-old male with no significant medical history who presented following a road traffic accident while driving at 60 km/hr without wearing a seat belt. The patient was not ejected from the vehicle and there was no rollover. He experienced a brief loss of consciousness. Advanced traumatic life support (ATLS) was initiated. During the primary survey, a superficial wound was noted on the anterolateral aspect of the right knee. In the secondary survey, there were evident deformity and tenderness in the right femur, although no open wounds were observed. Chest X-ray revealed multiple rib fractures from the 3rd to 6th ribs. Pelvic X-ray showed a right subtrochanteric femur fracture, which was further identified as a segmented fracture upon subsequent imaging. PAN CT was done and demonstrated unremarkable results. The knee wound was sutured, and the patient received analgesia, antibiotics, and prophylactic anticoagulation.

Three days later, the patient was transferred to our hospital. Upon arrival, the patient denied experiencing headache, loss of consciousness, vomiting, chest pain, shortness of breath, cough, or urinary symptoms. On examination, he was alert, conscious, oriented, and vitally stable. He presented with swelling and deformity of the right femur, with no open wounds or ecchymosis. Tenderness was noted over the middle shaft of the femur, with a reduced range of motion at the knee and hip due to pain. Distal neurovascular examination was intact, and the compartment was soft. Log roll examination revealed no abnormalities. The patient continued to receive analgesics, antibiotics, and prophylactic anticoagulation.

**Figure 1:**
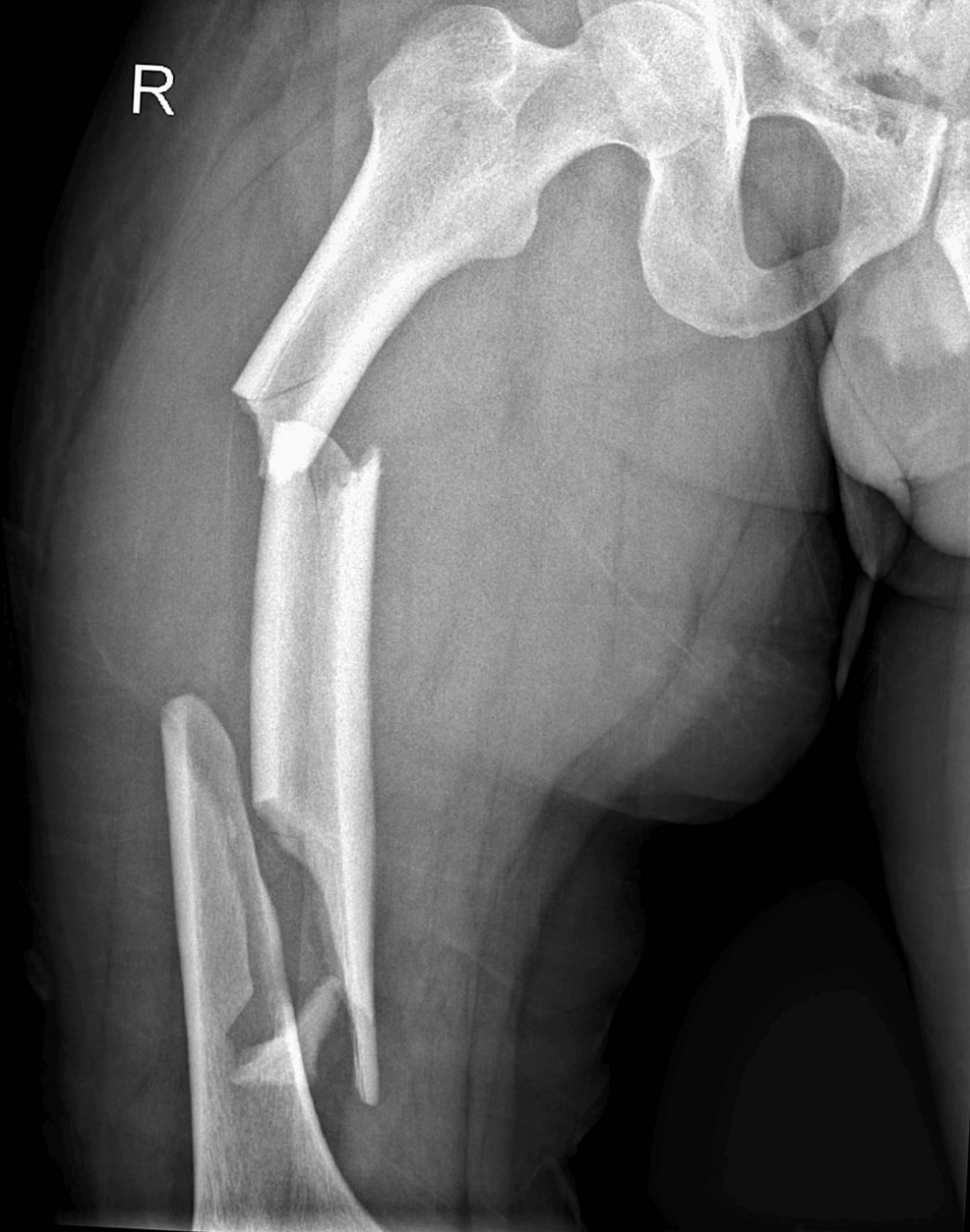
Pre-operative X-ray showing a segmented right femoral shaft (anteroposterior view of the right femur)

**Figure 2:**
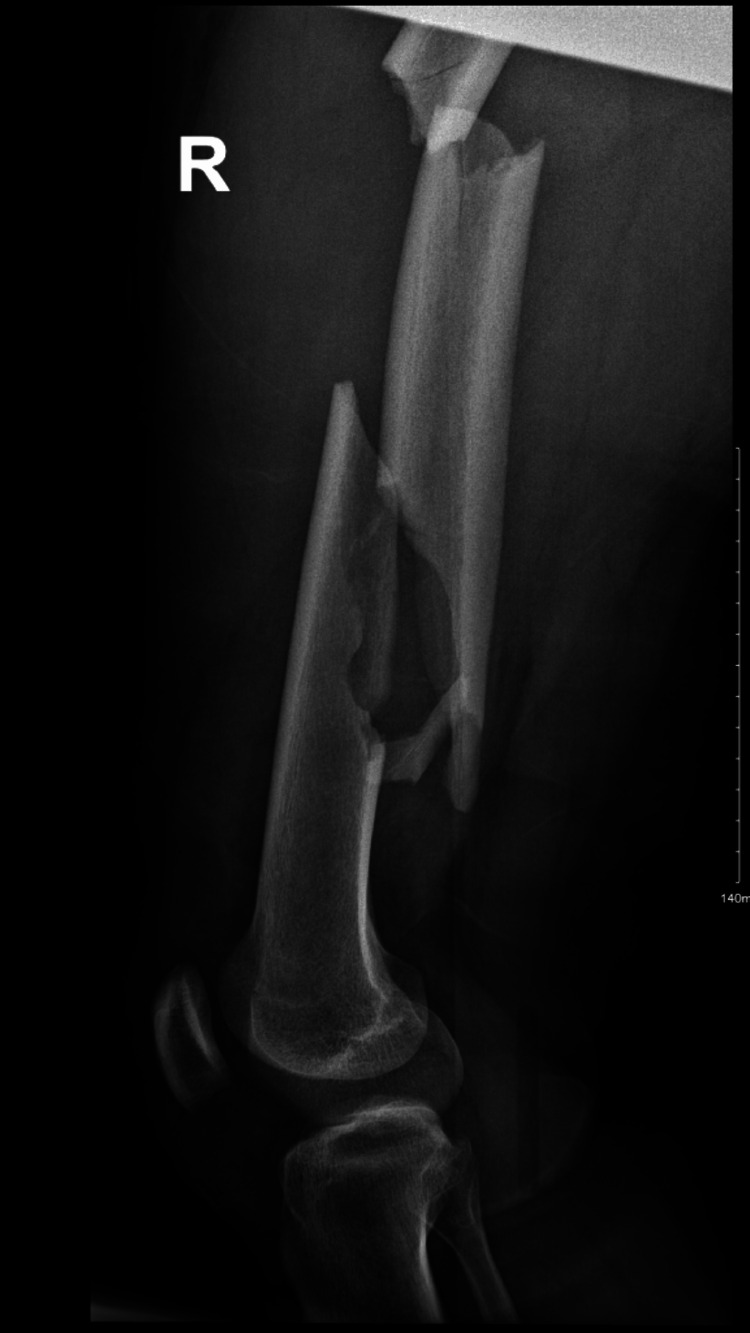
Pre-operative X-ray showing segmented right femoral shaft (lateral view of the right femur)

X-rays confirmed a segmented and comminuted fracture of the right femur shaft (Figures 1, 2). The patient was placed on skin traction, and thoracic surgery was consulted for the rib fractures. Subsequently, the patient underwent surgery, with consent obtained for closed or open reduction and internal fixation using an intramedullary nail for the right femur, along with wound irrigation and debridement.

Procedure details

Following the patient's transfer to the operating theater and completion of team briefing, general anesthesia was induced by the anesthesiologist. The patient was positioned on the traction table after ensuring proper fixation of the endotracheal tube. Preparatory measures included washing and shaving the right lower limb. The superficial knee wound on the anterolateral aspect, adjacent to the patella, was sutured using silk. An abrasion with superficial necrotic tissue was noted on the proximal tibia, which was subsequently washed.

Sterile preparation and draping were performed in the usual manner for femur fracture surgery. A 3 cm incision was made under C-arm guidance, proximal to the greater trochanter, and extended from the level of the anterior superior iliac spine towards the greater trochanter. Subcutaneous tissue and fascia were opened to access the fracture site. Under C-arm guidance, the entry point in the greater trochanter was identified using a pin. The pin was advanced approximately 6 cm into the proximal femur, followed by reaming to 40 mm. A reduction guide was utilized to align the fracture, particularly at the subtrochanteric level.

At the distal end, a long oblique supracondylar fracture with comminution was identified. An incision was made over this fracture, and cerclage was applied to stabilize the segment. Reduction clamps were used to hold the displaced butterfly fragment, followed by passage of the guide up to the knee to ensure correct length. Reaming continued, with attention to maintaining reduction at both the subtrochanteric and supracondylar fracture sites.

A decision was made to use a 11.5 x 40 META-TAN trochanteric antegrade nail due to the unavailability of a 42 cm length nail. A lag screw was placed in the proximal fracture after addressing external rotation issues. Distal interlocking screws were inserted, with one dynamic and one static screw used due to hardware availability constraints. The distal interlocking hole was challenging to navigate, necessitating a change in direction to avoid the medial femoral condyle.

Closure involved layer-by-layer suturing of fascia and subcutaneous tissue at various sites, including the entry point and fracture openings. The distal interlocking screw was secured through the supracondylar area. The patient's wounds were dressed, and he was extubated and transferred to the recovery room in stable condition.

**Figure 3:**
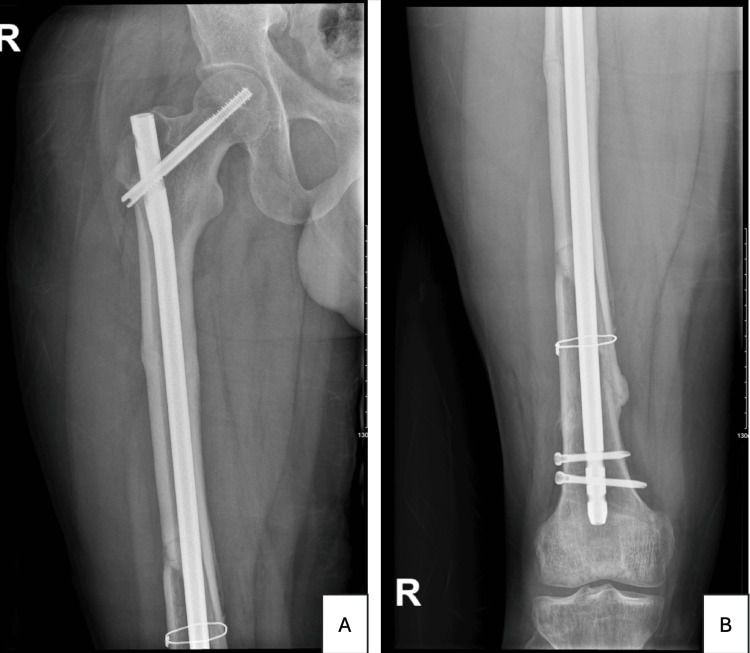
Post-operative radiographs demonstrating the anteroposterior view of the right femur (A and B)

Post-operatively, the patient continued to receive analgesia and prophylactic anticoagulation. Follow-up laboratory studies and X-rays were ordered to monitor progress and ensure optimal recovery (Figure 3).

Post-operation

The patient demonstrated an unremarkable examination post-operatively, including a distal neurovascular examination, which was intact. The patient tolerated the procedure very well.

Two days after the operation, he complained of bilateral foot heaviness and numbness. Later, the patient developed right foot drop with an inability to dorsiflex his ankle. Lumbar X-ray and CT of the knee were ordered to exclude occult fractures. Investigations showed an L5 lumbar pars defect and fractures of the right fibula and the posterior lateral aspect of the tibia. The first impression was right common peroneal nerve palsy (neuropraxia) due to the fibular fracture, exacerbated by intraoperative manipulation during femur fixation. Therefore, the patient underwent right lower limb electromyography and a nerve conduction study, which showed axonal injury to the right sciatic nerve with involvement of the common peroneal and tibial nerve fibers. Furthermore, the study showed bilateral absent sural nerve responses.

After that, the patient was followed in the clinic periodically. In the seventh week post-op, dorsiflexion started to improve with physiotherapy but had not fully recovered. The patient also developed erectile dysfunction since the surgery, yet he did not complain until the seventh week post-operatively. He was referred to urology for investigations and treatment. His hormonal profile, including FSH, LH, prolactin, and testosterone, was within the normal range. The patient was prescribed phosphodiesterase inhibitors for a month. After that, erection started to improve and recovered completely within six months of the operation. The 12-month follow-up of the patient demonstrated full recovery of dorsiflexion. The sensation improved, yet there was residual numbness only in the sole of the right foot. Right hip X-ray was ordered and showed remarkable healing of the fracture.

## Discussion

Femoral shaft fractures are among the most common injuries treated by orthopedic surgeons, typically resulting from high-energy trauma, often seen in motor vehicle accidents [3]. These fractures are mostly associated with other life-threatening injuries requiring the activation of the ATLS protocol initially to prioritize saving life over the limb. Furthermore, the definitive management of femoral shaft fractures is surgical fixation with intramedullary nailing (IMN) [4]. IMN procedures are performed with the patient in a supine position using a traction table, which aids in maintaining the correct position during fracture reduction. Traction tables offer several benefits, including improved acquisition of intraoperative radiographic images, enhanced stability while manipulating the fracture, and increased traction force [5]. Although traction tables have several advantages, they are also associated with certain complications often resulting from increased traction time or improper positioning. Complications associated with traction tables include neurological injuries, soft tissue damage, and fracture malrotation [5].

Our case developed two uncommon complications we believe are traction-table related. These complications included temporary erectile dysfunction and ipsilateral foot drop. The pudendal nerve is responsible for maintaining erection. Prolonged perineal traction during the fixation of femur fractures could cause ischemia in the responsible nerves leading to pudendal nerve neuropraxia which in turn causes erectile dysfunction [6]. This complication has been previously reported in multiple cases. In 1998, Lecumberri et al. reported a case of erectile dysfunction after the use of a traction table in an orthopedic procedure which spontaneously recovered after six months [7]. Moreover, Rajbabu et al. presented four cases of femur fractures that underwent internal fixation with a traction table and developed transient erectile dysfunction. All cases were eventually treated with phosphodiesterase inhibitors as in our case [8]. In addition, a systematic review that assessed the complications related to perineal post found 35 cases who developed erectile dysfunction [9]. Sciatic nerve injury after the fixation of femur fracture, on the other hand, has several etiologies. It could be due to the entrapment of the nerve as a result of the fracture, or due to hematoma formation post-operatively [10,11]. However, Alzahrani et al. reported ipsilateral foot drop post femur IMN related to the traction table similar to our case which made us consider mispositioning of the fracture table as a reasonable cause [12]. Finally, these complications can be addressed by decreasing procedure time, enhancing the position of the patient, and providing sufficient padding of the perineal post. Additionally, ongoing monitoring and adjustments during the procedure can further help in preventing such issues [5].

## Conclusions

In conclusion, while complications like erectile dysfunction and foot drop arising from mispositioning or prolonged compression on traction tables are infrequently reported, their significance mandates heightened awareness within the orthopedic community. Identification of predisposing factors and recognition by orthopedic surgeons are paramount for decreasing their incidence. Adherence to precise patient positioning protocols, ongoing education, and implementation of standardized guidelines stand as cornerstone strategies in avoiding these rare yet consequential complications.
